# Maternal Exposure of a Beetle to Pathogens Protects Offspring against Fungal Disease

**DOI:** 10.1371/journal.pone.0125197

**Published:** 2015-05-04

**Authors:** Joanna J. Fisher, Ann E. Hajek

**Affiliations:** Department of Entomology, Cornell University, Ithaca, New York, United States of America; CEFE, FRANCE

## Abstract

Maternal exposure to an immune challenge can convey enhanced immunity to invertebrate offspring in the next generation. We investigated whether maternal exposure of the Asian longhorned beetle, *Anoplophora glabripennis*, to two species of the fungus *Metarhizium* or the bacterium *Serratia marcescens* elicited transgenerational immune priming (TGIP). We tested specificity of this protection and whether occurrence of TGIP was dependent on maternal exposure to living versus dead pathogens. Our results show that TGIP occurred and protected offspring against *Metarhizium brunneum*. Maternal exposure to *S*. *marcescens* provided non-specific protection to offspring against a fungal pathogen, but TGIP in response to *Metarhizium* only occurred when offspring were exposed to the same fungal species that was used to prime mothers. Moreover, TGIP in response to *M*. *brunneum* occurred only after maternal exposure to living rather than dead fungus. Our findings suggest that occurrence of TGIP could be both specific and dependent on whether the pathogen was alive.

## Introduction

In invertebrates, exposure to an immune challenge (a pathogen or pathogen- derived factor) can enhance the immune response to a subsequent challenge, a phenomenon known as immune priming [1]. Enhanced immunocompetence can persist throughout development within individuals and can even be conveyed to offspring in the next generation, an effect called trans-generational immune priming (TGIP) [2–6]. Parental exposure to bacteria and bacterial cell wall components [3–4, 7–14], a DNA virus [2, 15], microsporidia [16] or fungal cell walls [2] can enhance offspring immunity and in some cases even offspring survival.

The degree to which a parental pathogen challenge provides specific protection (i.e., offspring are protected against only the same pathogen experienced by the parent) versus non-specific protection (i.e., offspring are protected against a pathogen not experienced by the parent) varies in different systems. Fungal pathogens used to challenge mothers provided non-specific protection to offspring against a virus [2] and a protist [16]. In contrast, in a different system offspring were only protected when exposed to the same bacteria experienced by the mother [11].

Few studies have taken into account whether exposure to a living pathogen is necessary for TGIP to occur. The majority of studies of TGIP in insects have been based on heat-killed pathogens injected into insect hosts, thereby bypassing the cuticular or gut defenses which a pathogen would normally have to overcome during the infection process. The use of heat-killed pathogens also prevents exposure of the insect to the cascades of pathogen-produced compounds including proteases and virulence factors characterizing a normal infection [17]. In *Drosophila* both fungal cell wall components and virulence factors are necessary to fully activate the host’s immune response against fungal entomopathogens through the Toll pathway which is involved in recognizing fungi and gram positive bacteria [18–20]. *Drosophila melanogaster* individuals mutant for the protease involved in detecting fungal virulence factors were more susceptible to fungal pathogens that secrete these virulence factors than flies that were able to detect these virulence factors [19]. Several studies have used living pathogens to prime insects within a generation [5, 7, 15–16, 21–22]. However, only two studies have investigated TGIP in response to living pathogens, including living virus [15] and living bacteria [9] but not living fungi, and we are aware of no studies directly comparing whether priming resulted from exposure to living versus dead pathogens.

We investigated TGIP in the Asian longhorned beetle, *Anoplophora glabripennis*, a wood borer native to China and Korea which has been introduced to Europe and northeastern North America [23–25]. This long-lived wood borer takes 1–2 years for larvae to develop and adults often live for more than a month. This beetle is under eradication everywhere that it has been introduced because it has the potential to cause extensive damage in urban and natural forests if it becomes established. The entomopathogenic fungus *Metarhizium brunneum* is pathogenic to *A*. *glabripennis* and is being developed as a means for biological control. We were interested in determining if a prior maternal pathogen exposure could make *M*. *brunneum* less effective in controlling *A*. *glabripennis* offspring.

We conducted bioassays to investigate 1.) whether maternal exposure to a living compared to a dead fungal pathogen (*M*. *brunneum*) conveys protection to *A*. *glabripennis* offspring, and 2.) the extent to which the primed response in offspring is specific to the pathogen experienced by mothers. We found variable specificity in TGIP in response to fungal versus bacterial pathogens. Additionally, only maternal exposure to a living fungal pathogen increased offspring resistance to that pathogen and exposure to heat-killed fungal pathogens did not elicit TGIP.

## Materials and Methods

### Insect culture

*Anoplophora glabripennis* were reared and tested in a quarantine facility (APHIS permit P526P-14-00458; Cornell University Sarkaria Arthropod Research Laboratory). Striped maple (*Acer pensylvanicum*) was used for adult food and oviposition. Detailed rearing protocols for adults and larvae are described in Ugine et al. [26]. Only adults were challenged with pathogens in this study. Prior to experiments, beetles were maintained at 14:10 h light:dark, with 25°C days and 16°C nights, individually in 473 ml lidded clear plastic cups containing striped maple twigs. The age of female beetles used for the maternal priming treatments averaged 7.7 ± 0.1 d (mean ± SE) post-eclosion at the beginning of the experiment. Offspring weight and whether or not offspring successfully completed development and eclosed as F1 generation adults were recorded. Mothers were exposed to either living *M*. *brunneum* or *M*. *anisopliae*, or to dead *M*. *brunneum* or *S*. *marcescens* and all offspring were challenged with *M*. *brunneum*.

### Fungal preparation

The F-52 isolate of *M*. *brunneum* (ARSEF 7711; USDA Agricultural Research Service Collection of Entomopathogenic Fungal Cultures, Ithaca, NY) and the closely related *M*. *anisopliae* isolate ESC1 (ATCC 32176; American Type Culture Collection, Manassas, VA) were grown at 26°C on potato dextrose agar (PDA) (Difco, Sparks, MD) with 0.1% gentamicin to control growth of contaminants.

Living fungal suspensions were prepared by washing conidia off of cultures using 0.05% Tween 80 and 2 mm glass balls to dislodge and suspend conidia. Conidial viability was quantified by spreading a 100 μl aliquot of 1 x 10^7^ conidia/ml onto each of 360-mm diam petri dishes containing PDA and incubating plates overnight at 26°C, after which they were examined to determine whether or not conidia were viable, indicated by the presence of germ tubes.

For treatment with a dead pathogen, conidia of *M*. *brunneum* were harvested from PDA plates and placed in an oven at 100°C for 1h, to kill the conidia, which were then harvested as above. The success of the heat-killed treatment was confirmed by plating the conidial suspension on PDA and incubating overnight at 26°C. The vaccine was prepared by suspending the heat-killed conidia in Grace’s Insect Media (GIM) (BioWhittaker, Walkersville, MD), and the suspension was then stored at -80°C until use.

### Bacterial preparation

*Serratia marcescens* was grown in Luria Bertani (LB) broth [27] overnight at ambient temperatures on a rotating shaker at 175 rpm. A portion of the inoculated broth was serially diluted, plated on Luria Bertani Agar (LA) and incubated overnight at 26°C to estimate colony forming units (CFUs)/ml. Bacteria in the remainder of the broth were heat-killed at 95–100°C for 20 min. The success of the heat-kill treatment was confirmed by plating the bacterial suspension on LA and incubating overnight at 26°C.

### Maternal inoculations with *M*. *brunneum* or *M*. *anisopliae*

Beetles that would become mothers were exposed to either living *M*. *brunneum* or *M*. *anisopliae* or dead *M*. *brunneum*. For exposures to living fungal pathogens, fungal suspensions used to inoculate beetles were adjusted to 10^3^ conidia/ml using 0.05% Tween 80. The doses of living fungi were limited by the virulence of the fungal pathogen because the experiment depended on the female beetles living long enough to reproduce. Females were inoculated by gently shaking them in 15 ml of a suspension of living conidia of either *M*. *brunneum* or *M*. *anisopliae* for 5 sec (solutions were vortexed for 15 sec prior to beetle inoculation). The average concentrations of viable conidia for *M*. *brunneum* and *M*. *anisopliae* suspensions were 9.56 × 10^2^ ± 1.44 × 10 conidia/ml and 9.63 × 10^2^ ± 5.61 × 10 conidia/ml, respectively, after accounting for conidial viability. This experiment was repeated 3 times, with *M*. *brunneum* (n = 5) and *M*. *anisopliae* (n = 5) inoculated female beetles in each replicate.

For treatment with the dead fungal pathogen, female beetles were injected with heat-killed *M*. *brunneum*. Female beetles were cold-anesthetized for 8 min, rinsed in 70% ethanol and injected with 10 μl of the heat-killed conidial suspension in the intersegmental membrane between the metasternum and the rear coxal cavity using a 3 ml syringe mounted in a syringe microburet (Micro-Metric Instrument Co, Cleveland, OH). The conidial suspension was vortexed for 15 sec before loading into the syringe. Control beetles were injected with 10 μl of sterile GIM. The average injected dose across replicates was 4.24 × 10^6^ ± 3.40 × 10^5^ conidia/ml (Rep 1: 4.03 × 10^6^ ± 1.28 × 10^6^; Rep 2: 3.78 × 10^6^ ± 1.64 × 10^5^; Rep 3: 4.90 × 10^6^ ± 4.63 × 10^5^ conidia/ml). The experiment was repeated 3 times with female *M*. *brunneum-*injected beetles (n = 5) and female GIM control beetles (n = 5) for each replicate.

### Maternal inoculations with *S*. *marcescens*

Female beetles were injected with 10 μl of 10^7^ CFU/ml heat-killed *S*. *marcescens* in sterile LB broth. Control beetles were injected with 10 μl of sterile LB broth. The experiment was repeated 3 times with *S*. *marcescens*-injected beetles (n = 5) and female broth control-injected beetles (n = 2) for each replicate.

### Rearing offspring

All treated females were mated with an untreated male beetle 6 d after pathogen inoculation. The one male that died during the experiment was incubated after death to confirm that it did not become infected with fungi acquired when mating with a fungal-treated female; the fungus did not sporulate from the cadaver, so it is unlikely that the male died of fungal infection. All pairs of beetles were maintained separately in 3.8 l glass jars containing 7 striped maple twigs and a bolt of striped maple and allowed to oviposit for 2 weeks. After each week, bolts were replaced. After 2 weeks males and females were removed from jars and maintained separately in 473 ml lidded clear plastic cups containing striped maple twigs and checked daily for mortality for 60 d.

### Offspring bioassays with living *M*. *brunneum*

Offspring hatching from eggs laid by treated females were reared to adult as described before. Adults from this F1 generation were challenged with living *M*. *brunneum* when used in offspring bioassays. The beetles averaged 22.3 ± 0.2 d post-eclosion at the start of the bioassays. Conidial viabilities were determined by applying a 10 μl aliquot of 10^7^conidia/ml cottonseed oil to a PDA plate and placing a coverslip on the drop to ensure that the conidia in oil contacted the agar. Using the resulting percent germination to quantify conidial viability, conidial suspensions were adjusted to 10^7^ viable conidia/ml using cottonseed oil.

Offspring from both the maternal treatment and control groups were inoculated by placing a 2 μl drop of 10^7^ conidia/ml on the inter-segmental membrane between the thorax and abdomen. Following inoculation the beetles were checked daily for death over 72 days. Bioassays were conducted 3 times with offspring from each maternal pathogen treatment (*S*. *marcescens* n = 62; heat-killed *M*. *brunneum* n = 87; living *M*. *brunneum* n = 51; living *M*. *anisopliae* n = 51; S1 Table in S1 File) and with offspring from each maternal control treatment (naive n = 60; broth injection n = 27; GIM injection n = 52). The 60 offspring from the naive maternal control treatment were used as untreated negative controls and received a drop of sterile cottonseed oil instead of being inoculated with *M*. *brunneum*.

### Data analysis

Bioassays ended at 72 days and living offspring were censored for analysis. Survival times for offspring were compared using proportional hazards models [28]. All non-significant interactions were removed from the models. The explanatory variables were replicate, sex, and maternal treatment. Survival curves and median days to death were calculated using Kaplan-Meier survival analyses.

Among offspring in bioassays, the times to death for beetles in each treatment were compared to the respective controls. If the treatment effect was significant then treatment levels were analyzed using risk ratios with alpha values adjusted using the Bonferroni correction to take into account multiple comparisons.

Failure of larvae and pupae to complete development and eclose was analyzed using a linear regression model. The proportion of offspring for each mother that failed to eclose was normalized using a log (x +1) transformation. The explanatory variables were replicate, maternal treatment, and female weight.

## Results

**Fig 1:**
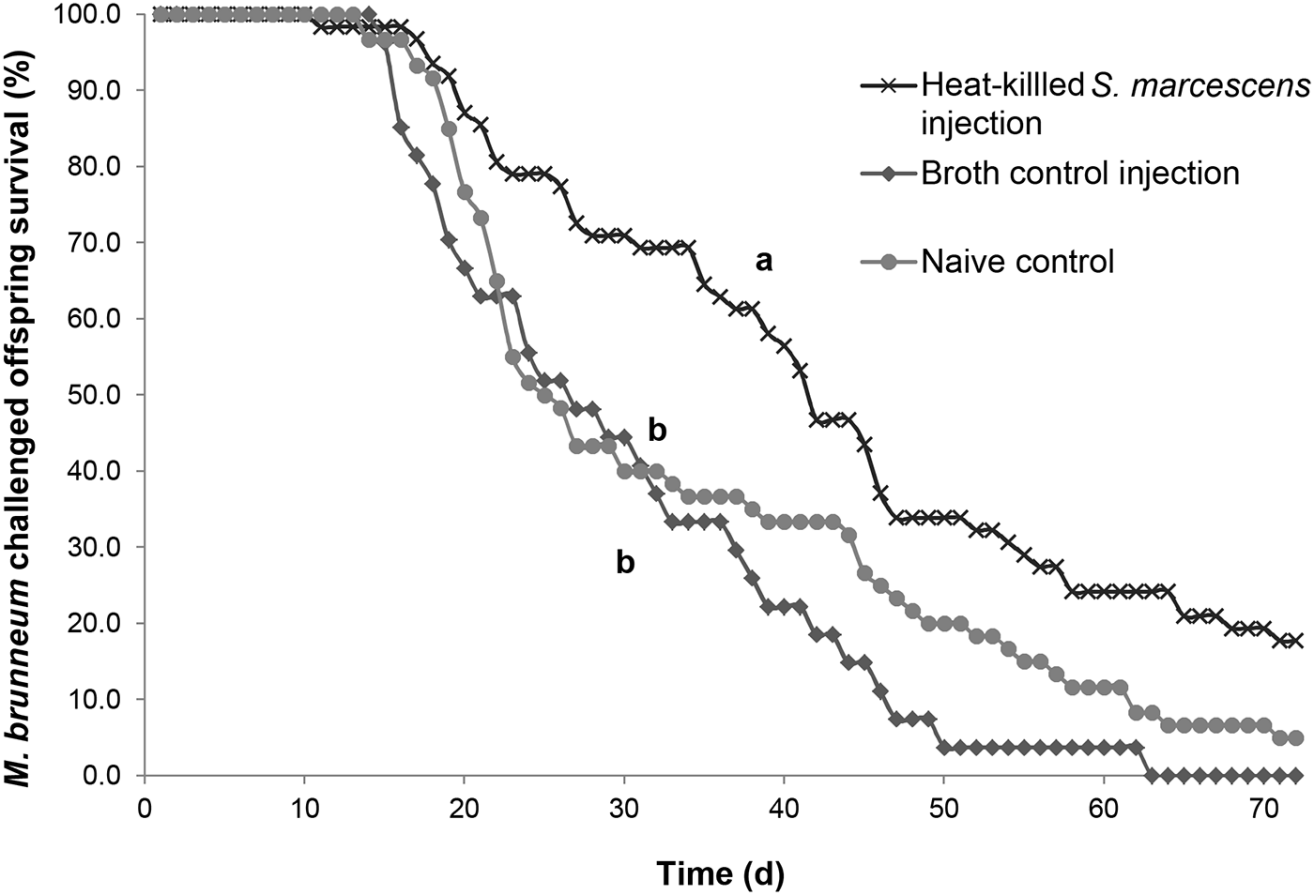
Survival curves for offspring of the *S*. *marcescens* treatment. Percentages of male and female (merged) offspring treated with *M*. *brunneum* surviving over time whose mothers were challenged with either *S*. *marcescens* or a control treatment (naive control or broth control). Different letters signify significant differences in survival curves between treatments (risk ratios, p ≤ 0.0167).

**Fig 2:**
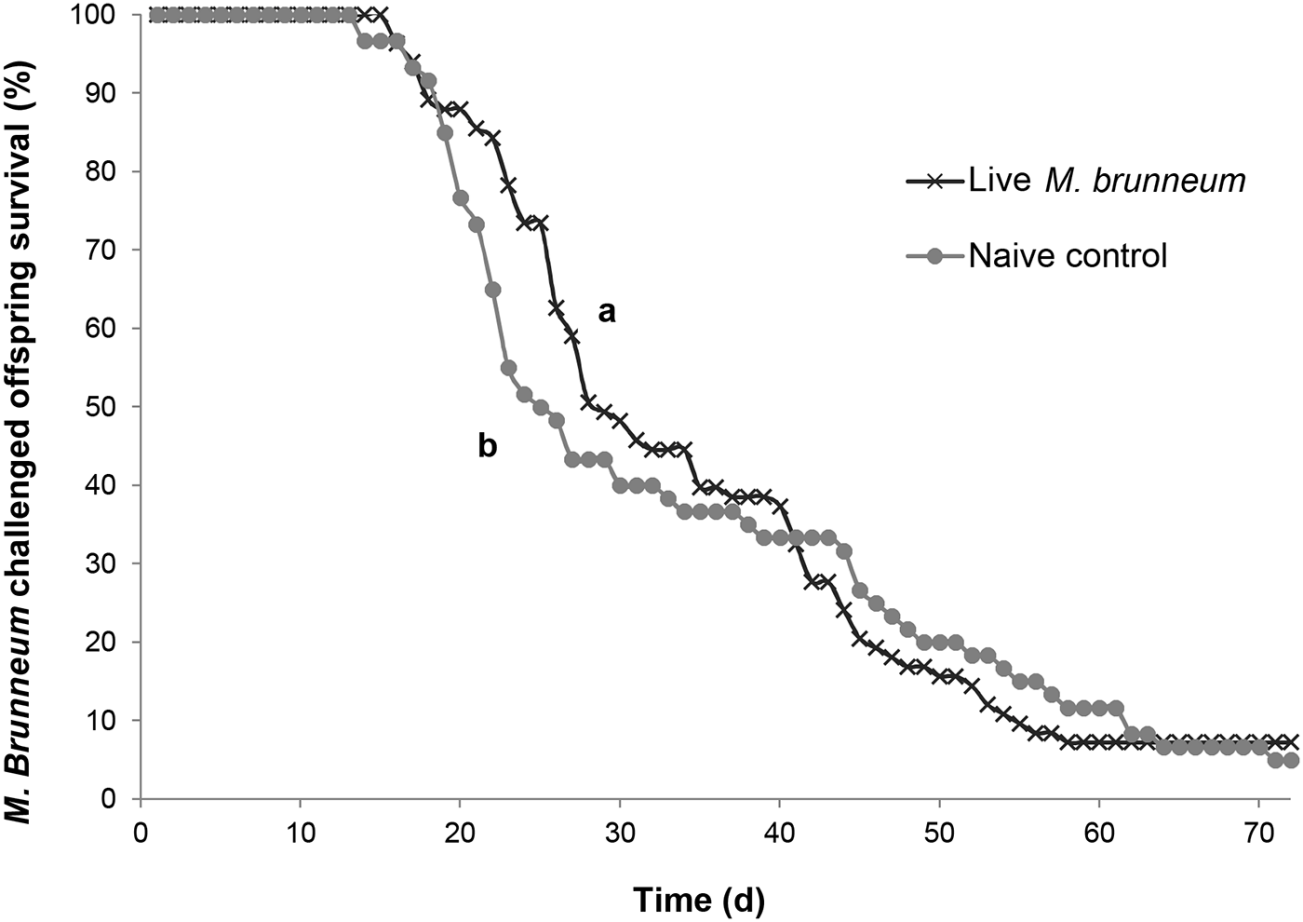
Survival curves for offspring of the live *M*. *brunneum* treatment. Percentages of male and female (merged) offspring treated with *M*. *brunneum* surviving over time whose mothers were challenged with either a living dose of *M*. *brunneum* or a control treatment (naive control). Different letters signify significant differences in survival curves between treatments (χ^2^_1_ = 9.07, p = 0.0026).

We found evidence of TGIP for offspring challenged with *M*. *brunneum* whose mothers were treated with the gram negative bacterium *S*. *marcescens*, as these offspring lived significantly longer than controls (χ^2^_2_ = 23.57, p < 0.0001; risk ratios, p ≤ 0.0167, Table 1, Fig 1). Maternal exposure to *S*. *marcescens* increased offspring survival by a median of 15–16.5 d compared to controls (median days to death: *S*. *marcescens*, 42 d (95% CI: 36, 46); naive control, 25.5 d (CI: 23, 34); broth control, 27 d (CI: 19, 37)). There was no effect of sex for time to death of offspring whose mothers were either exposed to *S*. *marcescens* or received a control treatment *(*χ^2^_1_ = 1.99, p = 0.1585). Additionally, we found evidence of immune priming for offspring challenged with *M*. *brunneum* whose mothers were treated with living *M*. *brunneum* (χ^2^_1_ = 9.07, p = 0.0026, Fig 2). These beetles lived significantly longer than naive controls (median days to death: living *M*. *brunneum*, 29 d (CI: 27, 37); naive control, 25.5 d (CI: 23, 34)). There was no effect of sex for offspring whose mothers were either exposed to living *M*. *brunneum* or received the control treatment (χ^2^_1_ = 0.22, p = 0.6363). Offspring challenged with *M*. *brunneum* whose mothers were injected with heat-killed *M*. *brunneum* or inoculated with living *M*. *anisopliae* did not live significantly longer than the respective controls (heat-killed *M*. *brunneum*, χ^2^_2_ = 0.74, p = 0.6896, S1 Fig; living *M*. *anisopliae*, χ^2^_1_ = 0.13, p = 0.7216, S2 Fig). There was no effect of sex for offspring whose mothers were exposed to heat-killed *M*. *brunneum* or offspring exposed to living *M*. *anisopliae* or their respective controls (heat-killed *M*. *brunneum*, χ^2^_1_ = 0.38, p = 0.5375; living *M*. *anisopliae*, χ^2^_1_ = 0.36, p = 0.5465). Interactions between sex and treatment for all models were not significant and were removed.

**Table 1:** Offspring (F1 generation) bioassay experimental design and results.

Primed	Offspring Challenge	Controls	Model	χ^2^	df	p-value	TGIP^1^?	Pair-wise comparisons	risk ratio	p-value α = 0.0167
Heat-killed *S*. *marcescens*	Living *M*. *brunneum*	Naive, Broth Injection	Treatment	23.57	2	< 0.0001	Yes	*S*. *marcescens* vs Naive	0.4726	0.0004
			Sex	1.99	1	0.1585		*S*. *marcescens* vs Broth Injection	0.2919	< 0.0001
			Rep	107.7	2	< 0.0001		Naive vs Broth Injection	0.6176	0.0478
Heat-killed *M*. *brunneum*	Living *M*. *brunneum*	Naive, GIM^2^ Injection	Treatment	0.74	2	0.6896	No			
			Sex	0.38	1	0.5375				
			Rep	144.8	2	< 0.0001				
Living *M*. *brunneum*	Living *M*. *brunneum*	Naive	Treatment	9.07	1	0.0026	Yes			
			Sex	0.22	1	0.6363				
			Rep	101.4	2	< 0.0001				
Living *M*. *anisopliae*	Living *M*. *brunneum*	Naive	Treatment	0.13	1	0.7216	No			
			Sex	0.36	1	0.5465				
			Rep	88.17	2	< 0.0001				

Statistics were generated using proportional hazards models and risk ratios adjusted using a Bonferroni correction for multiple comparisons (α = 0.0167).

^1^TGIP = Transgenerational Immune Priming,

^2^Grace's Insect Media.

There was no effect of maternal treatment or weight on whether or not offspring failed to complete development and eclose (treatment F_5, 69_ = 0.40, *p* = 0.85; female weight F_1, 69_ = 1.19, *p* = 0.28).

## Discussion

We found that TGIP was dependent on whether mothers were exposed to a living or dead fungal pathogen, i.e., offspring of mothers exposed to living but not heat-killed *M*. *brunneum* lived longer than controls when challenged with *M*. *brunneum*. Only two other studies have investigated TGIP with a fungal pathogen. Priming in mosquitoes was found in response to living fungi [16], as is consistent with our findings. However, Huang and Song [2] found TGIP in shrimp in response to glucans, which are components of all fungal cell walls. Glucans would have been present in the cell walls of heat-killed *M*. *brunneum* but we did not find priming in response to heat-killed *M*. *brunneum*. Although these former studies found TGIP in response to living or dead pathogens, we are aware of no other studies that have made a direct comparison between a living versus dead challenge with the same pathogen species.

We hypothesize that heat-killed *M*. *brunneum* did not induce TGIP because it lacked the compounds produced during an active fungal infection. There is some evidence that substances produced during pathogen growth are necessary for priming to occur, at least within a generation. For example, Milutinović et al. [29] found that for larvae of the flour beetle *Tribolium castaneum*, challenged with the gram positive bacterium *Bacillus thuringiensis*, priming within a single generation only occurred when larvae were exposed to spore supernatants containing substances produced during bacterial growth. Priming however did not occur when larvae were exposed to autoclaved or heat-killed pathogen cells that would have lacked these substances [29].

In our study, it is also possible that differences in the methods used for inoculation with living versus dead pathogens could have influenced results. The heat-killed pathogens were injected into the insects’ hemocoel, while the living fungal pathogens were applied externally to the beetles’ cuticle. The heat-killed pathogen was injected to ensure the pathogen would elicit the insects’ immune response as these insects exhibit very limited grooming behavior [JJF unpublished data] so they would not become inoculated with topical application. In contrast, the living fungal pathogen was applied externally because if this pathogen was injected, insects would have been killed before they could reproduce. The insect cuticle provides not only mechanical protection but is also an active biochemical barrier with antibacterial compounds and the ability to inhibit the growth of fungal germ tubes before they enter the hemocoel [30]. Therefore it is possible that differences in time to death among treatments with living versus dead pathogens could be influenced in part by the fact that heat-killed fungi and bacteria were injected into the insect and bypassed this defensive barrier while living fungal pathogens had to enter through the cuticle.

Maternal exposure to fungi only provided specific protection when both mothers and offspring were exposed to the same isolate of *M*. *brunneum*, but not when mothers were exposed to *M*. *anisopliae* and offspring were challenged with *M*. *brunneum*. A similar specific TGIP response was also seen in *T*. *castaneum* exposed to bacteria [11]. Offspring of either paternally or maternally primed *T*. *castaneum* adults had enhanced survival when exposed to the same bacterial challenge as their parent but not when exposed to a different bacterium. Inducing a specific transgenerational immune response would be advantageous if the next generation will encounter the same pathogen as the parental generation and specificity would be advantageous in this circumstance as immune priming can be costly for both mothers and offspring [11, 13, 31–33].

Maternal immune priming can result in reductions in maternal and offspring fecundity, slower development time in offspring or decreased offspring developmental time resulting in a decrease in pupal weight [11, 13, 31–33]. The potential costs that can be associated with priming make it especially important that TGIP provides protection to offspring against pathogens they are likely to encounter. However, in this study we saw no effect of maternal treatment on the ability of offspring to eclose, regardless of treatment, although it is possible that there are other costs present in this system which we did not investigate. Our findings agree with those of Freitak et al. [7] and Zanchi et al. [13] who also found no decrease in offspring survival to adulthood after maternal exposure to a bacterial challenge.

Unlike maternal exposure to fungal pathogens, maternal exposure to heat-killed *S*. *marcescens* provided non-specific protection to offspring challenged with *M*. *brunneum*. However it is not known whether exposure to heat-killed *S*. *marcescens* would provide specific protection to offspring because offspring were not challenged with *S*. *marcescens*. TGIP conveying non-specific protection to offspring has been observed in other systems as well [2, 16]. Thus, we found variable specificity in TGIP against two entomopathogens in the fungal genus *Metarhizium* and maternal exposure to the gram negative bacterium *S*. *marcescens* protected offspring against a fungal pathogen. In *Drosophila* fungi and gram negative bacteria induce different pathways in the insect immune response. Gram negative bacteria primarily induce the IMD pathway while fungi primarily induce the Toll pathway [34]. However in *T*. *castaneum* both a gram positive and a gram negative bacterial species as well as fungus induced both the Toll and IMD pathways [35]. This demonstrated that the induction of these pathways by microbes is more promiscuous in *T*. *castaneum* than in *Drosophila*. It may be that heat-killed *S*. *marcescens* led to TGIP because of the cross-talk between the Toll and IMD pathways in coleopteran insects. It could be that heat-killed *S*. *marcescens* provided greater protection to offspring than *M*. *brunneum* because of differences in how these pathogens induce the insect’s immune response.

## Conclusions

We found evidence that maternal exposure to a living compared to a dead fungal pathogen was necessary to convey protection to offspring of *A*. *glabripennis*. We speculate this was because the heat-killed *M*. *brunneum* lacked the compounds normally produced during fungal infection that would induce priming. We also found that offspring were only protected with maternal exposure to the same fungal pathogen that was experienced by the offspring and maternal exposure to *S*. *marcescens* bacteria provided non-specific protection to offspring against a fungal pathogen.

## Supporting Information

S1 Fig Survival curves for offspring of the heat-killed *M*. *brunneum* treatment. Percentages of male and female (merged) offspring treated with *M*. *brunneum* surviving over time whose mothers were challenged with either heat-killed *M*. *brunneum* or a control treatment (naive control or GIM injection). There were no significant differences between treatment survival curves (χ^2^_2_ = 0.74, p = 0.6896).
(TIF)

S2 Fig Survival curves for offspring of the live *M*. *anisopliae* treatment. Percentages of male and female (merged) offspring treated with *M*. *brunneum* surviving over time whose mothers were challenged with either a living dose of *M*. *anisopliae* or a control treatment (naive control). There were no significant differences between treatment survival curves (χ^2^_1_ = 0.13, p = 0.7216).
(TIF)

S1 File File includes S1 Table. S1 Table: Sample sizes for offspring bioassay. Number of offspring from the maternal treatment and control groups inoculated with *M*. *brunneum* and untreated offspring used as negative controls for each experimental replicate.
(DOCX)

## References

[1] Kurtz J, Armitage SA. Alternative adaptive immunity in invertebrates. Trends Immunol. 2006; 27: 493–496. 16979938 10.1016/j.it.2006.09.001

[2] Huang C-C, Song Y-L. Maternal transmission of immunity to white spot syndrome associated virus (WSSV) in shrimp *Penaeus monodon* . Dev Compar Immunol. 1999; 23: 545–552. 10579383 10.1016/s0145-305x(99)00038-5

[3] Hernández López J, Schuehly W, Crailsheim K, Riessberger-Gallé U. Trans-generational immune priming in honeybees. Proc R Soc B. 2014; 281; 20140454 10.1098/rspb.2014.0454 24789904 PMC4024302

[4] Moret Y, Schmid-Hempel P. Entomology: Immune defense in bumble-bee offspring. Nature. 2001; 414: 506 11734840 10.1038/35107138

[5] Reber A, Chapuisat M. No evidence for immune priming in ants exposed to a fungal pathogen. PLoS ONE. 2012; 7: e35372 10.1371/journal.pone.0035372 22523588 PMC3327680

[6] Thomas AM, Rudolf VHW. Challenges of metamorphosis in invertebrate hosts: maintaining parasite resistance across life-history stages. Ecol Entomol. 2010; 35: 200–205.

[7] Freitak D, Heckel DG, Vogel H. Dietary-dependent trans-generational immune priming in an insect herbivore. Proc R Soc B. 2009; 276: 2617–2624. 10.1098/rspb.2009.0323 19369263 PMC2686660

[8] Freitak D, Schmidtberg H, Dickel F, Lochnit G, Vogel H, Vilcinskas A. The maternal transfer of bacteria can mediate trans-generational immune priming in insects. Virulence. 2014; 5: 547–554. 10.4161/viru.28367 24603099 PMC4063815

[9] Little TJ, O’Connor B, Colegrave N, Watt K, Read AF. Maternal transfer of strain-specific immunity in an invertebrate. Curr Biol. 2003; 13: 489–492. 12646131 10.1016/s0960-9822(03)00163-5

[10] Moret Y. ‘Trans-generational immune priming’: Specific enhancement of the antimicrobial immune response in the mealworm beetle, *Tenebrio molitor* . Proc R Soc B. 2006; 273: 1399–1405. 16777729 10.1098/rspb.2006.3465 PMC1560290

[11] Roth O, Joop G, Eggert H, Hilbert J, Daniel J, Schmid-Hempel P, et al Paternally derived immune priming for offspring in the red flour beetle, *Tribolium castaneum* . J Anim Ecol. 2010; 79: 403–413. 10.1111/j.1365-2656.2009.01617.x 19840170

[12] Sadd BM, Kleinlogel Y, Schmid-Hempel R, Schmid-Hempel P. Trans-generational immune priming in a social insect. Biol Lett 1. 2005; 386–388. 10.1098/rsbl.2005.0369 PMC1626361 17148213

[13] Zanchi C, Troussard J-P, Martinaud G, Moreau J, Moret Y. Differential expression and costs between maternally and paternally derived immune priming for offspring in an insect. J Anim Ecol 80. 2011; 1174–1183. 10.1111/j.1365-2656.2011.01872.x 21644979

[14] Zanchi C, Troussard J-P, Moreau J, Moret Y. Relationship between maternal transfer of immunity and mother fecundity in an insect. Proc R Soc B. 2012; 279: 3223–3230. 10.1098/rspb.2012.0493 22535782 PMC3385725

[15] Tidbury HJ, Pedersen AB, Boots M. Within and transgenerational immune priming in an insect to a DNA virus. Proc R Soc B. 2011; 278: 871–876. 10.1098/rspb.2010.1517 20861049 PMC3049047

[16] Lorenz LM, Koella JC. Maternal environment shapes the life history and susceptibility to malaria of *Anopheles gambiae* mosquitoes. Malar J. 2011; 10: 382 10.1186/1475-2875-10-382 22188602 PMC3269443

[17] Vega FE, Lacey LA. Insect Pathology (2nd Ed.). New York: Academic Press 2012; pp. 27–72.

[18] Chamy LE, Leclerc V, Caldelari I, Reichhart J-M. Sensing of “danger signals” and pathogen-associated molecular patterns defines binary signaling pathways “upstream” of Toll. Nature Immunol. 2008; 9: 1165–1170. 10.1038/ni.1643 18724373 PMC2768518

[19] Gottar M, Gobert V, Matskevich AA, Reichhart J-M, Wang C, Butt TM, et al Dual detection of fungal infections in *Drosophila* via recognition of glucans and sensing of virulence factors. Cell. 2006; 127: 1425–1437. 17190605 10.1016/j.cell.2006.10.046 PMC1865096

[20] Valanne S, Wang J-H, Rämet M. The *Drosophila* Toll signaling pathway. J Immunol. 2011; 186: 649–656. 10.4049/jimmunol.1002302 21209287

[21] Kurtz J, Franz K. Innate defence: Evidence for memory in invertebrate immunity. Nature. 2003; 425: 37–38. 12955131 10.1038/425037a

[22] Rodrigues J, Brayner FA, Alves LC, Dixit R, Barillas-Mury C. Hemocyte differentiation mediates innate immune memory in *Anopheles gambiae* mosquitoes. Science. 2010; 329: 1353–1355. 10.1126/science.1190689 20829487 PMC3510677

[23] Brabbs T, Collins D, Hérard F, Maspero M, Eyre D. Prospects for the use of biological control agents against *Anoplophora* in Europe. Pest Manag Sci. 2015; 71: 7–14. 10.1002/ps.3907 25216358

[24] Haack RA, Bauer LS, Gao R, McCarthy JJ, Miller DL, Petrice TR, et al *Anoplophora glabripennis* within-tree distribution, seasonal development, and host suitability in China and Chicago. Gt Lakes Entomol. 2006; 39: 169.

[25] Hu J, Angeli S, Schuetz S, Luo Y, Hajek AE. Ecology and management of exotic and endemic Asian longhorned beetle *Anoplophora glabripennis* . Agric For Entomol. 2009; 11: 359–375.

[26] Ugine TA, Gardescu S, Hajek AE. The effect of exposure to imidacloprid on Asian longhorned beetle (Coleoptera: Cerambycidae) survival and reproduction. J Econ Entomol. 2011; 104: 1942–1949. 22299356 10.1603/ec11139

[27] Klein M. Bacteria of soil-inhabiting insects In: Lacey L, editor. Manual of techniques in insect pathology. New York: Academic Press 1997; pp. 101–115.

[28] SAS Institute. JMP, version 9.0.02. SAS Institute, Cary, NC 2010.

[29] Milutinović B, Fritzlar S, Kurtz J. Increased survival in the red flour beetle after oral priming with bacteria-conditioned media. J Innate Immun. 2014; 6: 306–314. 10.1159/000355211 24216503 PMC6742957

[30] Moret Y, Moreau J. The immune role of the arthropod exoskeleton. Invert Surviv J. 2012; 9: 200–206.

[31] Contreras-Garduño J, Rodríguez MC, Rodríguez MH, Alvarado-Delgado A, Lanz-Mendoza H. Cost of immune priming within generations: trade-off between infection and reproduction. Microb Infect. 2014; 16: 261–267. 10.1016/j.micinf.2013.11.010 24291714

[32] Sadd BM, Schmid-Hempel P. A distinct infection cost associated with trans-generational priming of antibacterial immunity in bumble-bees. Biol Lett. 2009; 5: 798–801. 10.1098/rsbl.2009.0458 19605389 PMC2827998

[33] Trauer U, Hilker M. Parental legacy in insects: Variation of transgenerational immune priming during offspring development. PLoS ONE. 2013; 8: e63392 10.1371/journal.pone.0063392 23700423 PMC3658988

[34] Lemaitre B, Hoffmann J. The host defense of *Drosophila melanogaster* . Annu Rev Immunol. 2007; 25: 697–743. 17201680 10.1146/annurev.immunol.25.022106.141615

[35] Yokoi K, Koyama H, Minakuchi C, Tanaka T, Miura K. Antimicrobial peptide gene induction, involvement of Toll and IMD pathways and defense against bacteria in the red flour beetle, *Tribolium castaneum* . Results Immunol. 2012; 2: 72–82. 10.1016/j.rinim.2012.03.002 24371569 PMC3862384

