# Supplementary figures and images for: Maternal Exposure of a Beetle to Pathogens Protects Offspring against Fungal Disease
Source: PLoS One. 2015 May 4;10(5):e0125197. doi: 10.1371/journal.pone.0125197 (PMC4418818; doi:10.1371/journal.pone.0125197)

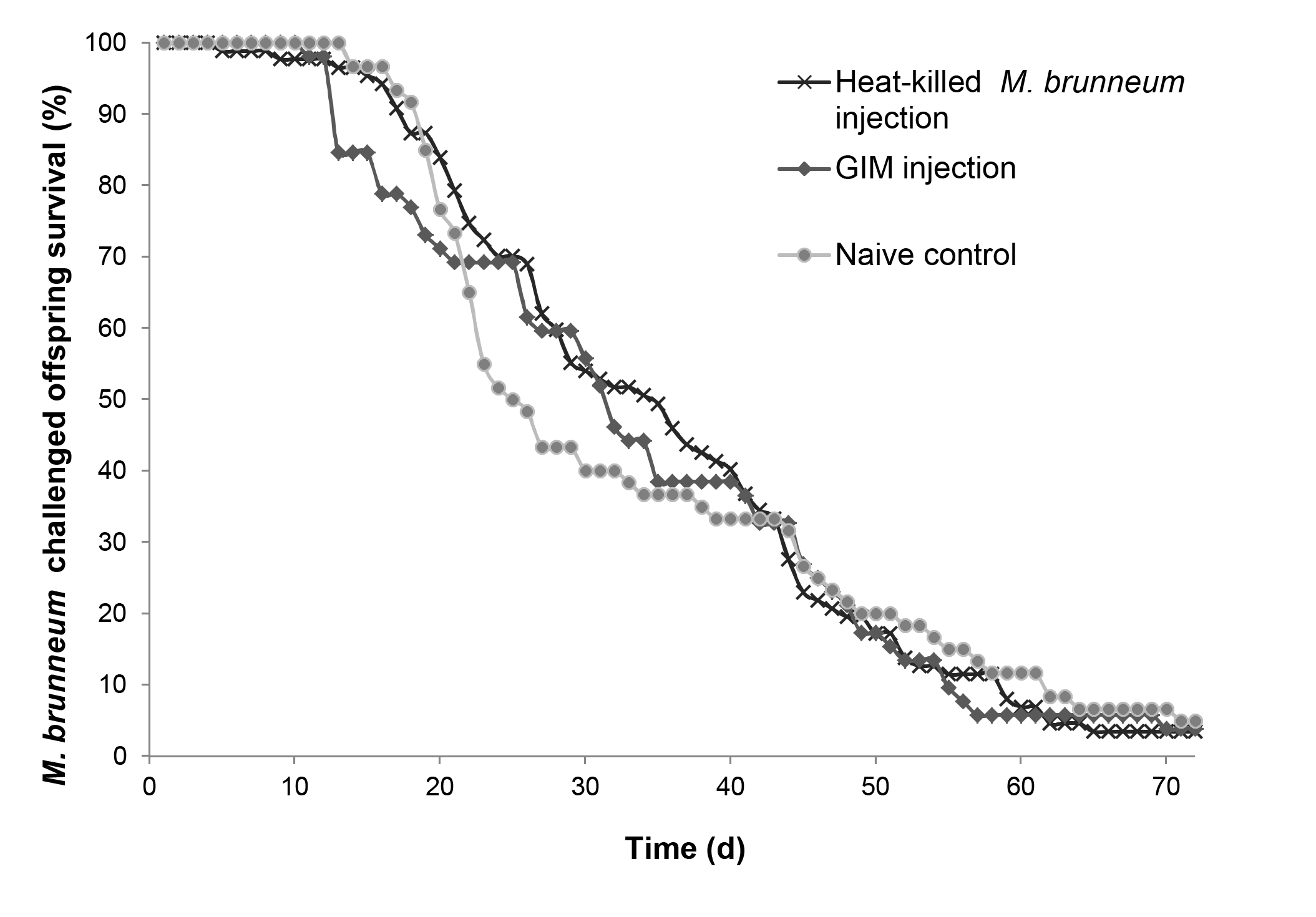

Supplement: S1 Fig — Percentages of male and female (merged) offspring treated with M. brunneum surviving over time whose mothers were challenged with either heat-killed M. brunneum or a control treatment (naive control or GIM injection). There were no significant differences between treatment survival curves (χ2 2 = 0.74, p = 0.6896). (TIF) [file pone.0125197.s001.tif]

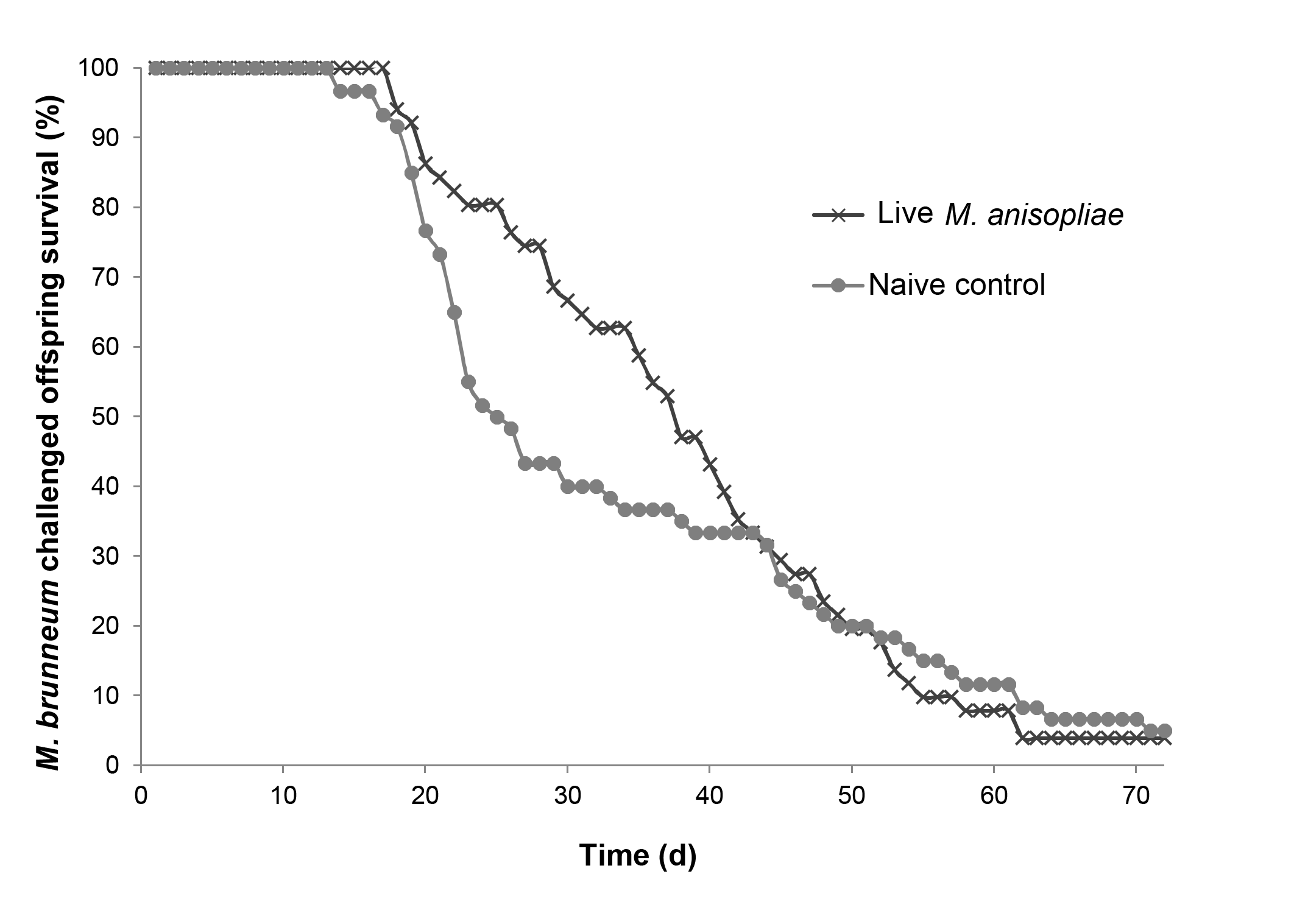

Supplement: S2 Fig — Percentages of male and female (merged) offspring treated with M. brunneum surviving over time whose mothers were challenged with either a living dose of M. anisopliae or a control treatment (naive control). There were no significant differences between treatment survival curves (χ2 1 = 0.13, p = 0.7216). (TIF) [file pone.0125197.s002.tif]
