# Supplementary material for: Maternal Exposure of a Beetle to Pathogens Protects Offspring against Fungal Disease
Source: PLoS One. 2015 May 4;10(5):e0125197. doi: 10.1371/journal.pone.0125197 (PMC4418818; doi:10.1371/journal.pone.0125197)
Supplement: S1 File — S1 Table: Sample sizes for offspring bioassay. Number of offspring from the maternal treatment and control groups inoculated with M. brunneum and untreated offspring used as negative controls for each experimental replicate. (DOCX) [file pone.0125197.s003.docx]

**Supporting Information**

**Table S1. Sample sizes for offspring bioassays.** Sample sizes for offspring from the maternal treatment and control groups inoculated with *M. brunneum* and untreated offspring used as negative controls.

**Figure Legends**

**Figure S1. Survival curves for offspring of the heat-killed *M. brunneum* treatment*.*** Percentages of male and female (merged) offspring treated with *M. brunneum* surviving over time whose mothers were challenged with either heat-killed *M. brunneum* or a control treatment (naive control or GIM injection). There were no significant differences between treatment survival curves (χ^2^_2_ = 0.74, p = 0.6896).

**Figure S2. Survival curves for offspring of the live *M. anisopliae* treatment.** Percentages of male and female (merged) offspring treated with *M. brunneum* surviving over time whose mothers were challenged with either a living dose of *M. anisopliae* or a control treatment (naive control). There were no significant differences between treatment survival curves (χ^2^_1_ = 0.13, p = 0.7216).
